# Uric acid and neurological disease: a narrative review

**DOI:** 10.3389/fneur.2023.1164756

**Published:** 2023-06-01

**Authors:** Naoyuki Otani, Eisei Hoshiyama, Motoshi Ouchi, Hidehiro Takekawa, Keisuke Suzuki

**Affiliations:** ^1^Department of Cardiology, Dokkyo Medical University Nikkyo Medical Center, Mibu, Japan; ^2^Department of Neurology, Dokkyo Medical University, Mibu, Japan; ^3^Department of Pharmacology and Toxicology, Dokkyo Medical University School of Medicine, Mibu, Japan; ^4^Stroke Center, Dokkyo Medical University, Mibu, Japan

**Keywords:** hyperuricemia, uric acid, neurological disease, stroke, neurodegenerative disease

## Abstract

Hyperuricemia often accompanies hypertension, diabetes, dyslipidemia, metabolic syndrome, and chronic renal disease; it is also closely related to cardiovascular disease. Moreover, several epidemiological studies have linked hyperuricemia and ischemic stroke. However, uric acid may also have neuroprotective effects because of its antioxidant properties. An association between low uric acid levels and neurodegenerative diseases has been suggested, which may be attributed to diminished neuroprotective effects as a result of reduced uric acid. This review will focus on the relationship between uric acid and various neurological diseases including stroke, neuroimmune diseases, and neurodegenerative diseases. When considering both the risk and pathogenesis of neurological diseases, it is important to consider the conflicting dual nature of uric acid as both a vascular risk factor and a neuroprotective factor. This dual nature of uric acid is important because it may help to elucidate the biological role of uric acid in various neurological diseases and provide new insights into the etiology and treatment of these diseases.

## 1. Introduction

**Figure 1:**
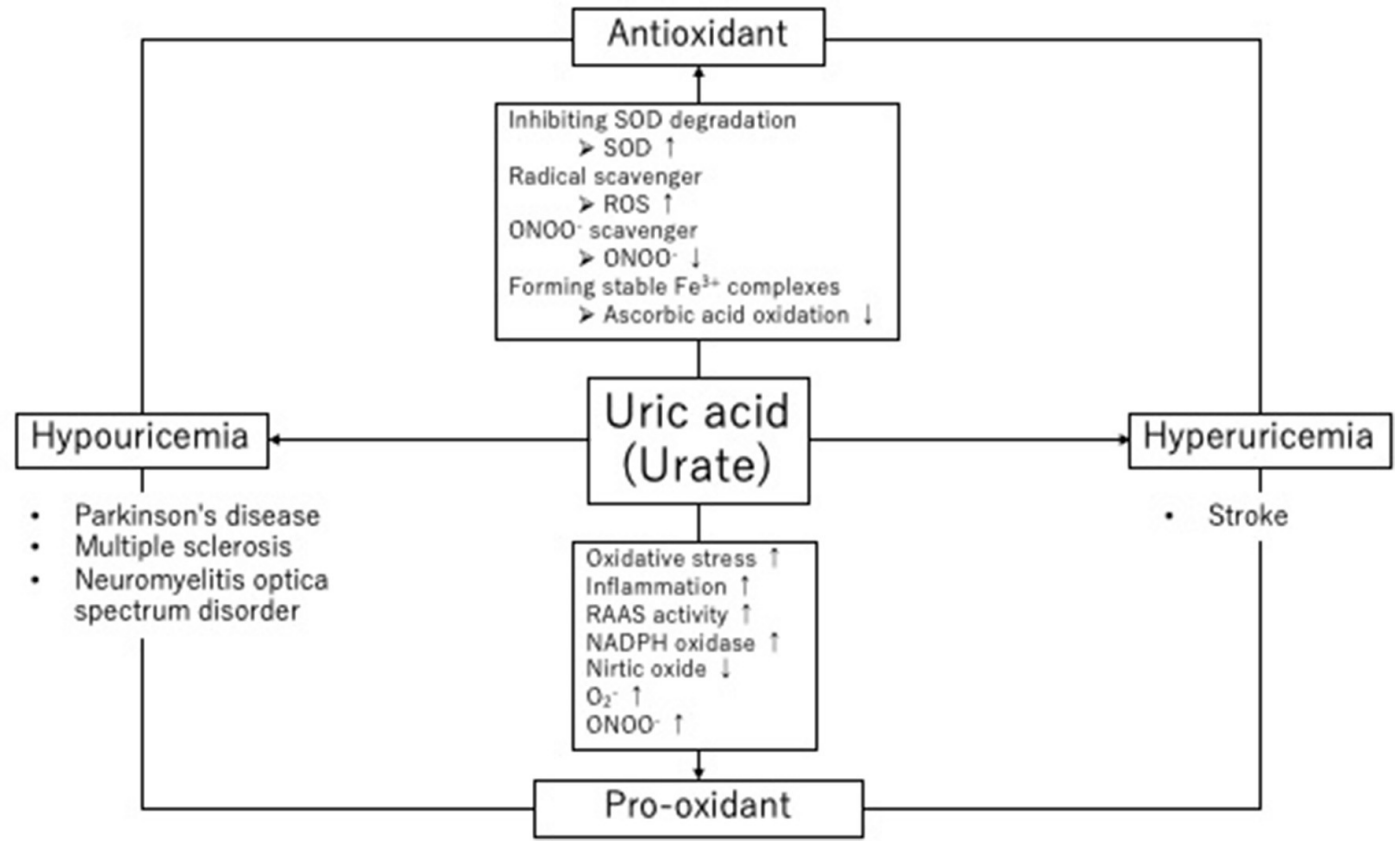
Role of uric acid in oxidative stress and its antioxidant properties. Uric acid has a dual nature, with both antioxidant and pro-oxidant effects, in neurological diseases. NO, nitric oxide; SOD, superoxide dismutase; ROS, radical oxygen species; ONOO^−^, peroxynitrite; RAAS, renin–angiotensin–aldosterone system; NADPH, nicotinamide adenine dinucleotide phosphate.

Serum uric acid levels are determined by the balance between uric acid production and its excretion by the kidneys and intestinal tract. When this balance is disrupted, both hyperuricemia and hypouricemia can occur. The upper limit of the reference value of serum uric acid levels for both sexes and all ages is 7.0 mg/dL; hyperuricemia is defined as exceeding this level (1). Lifestyle-related diseases such as obesity, metabolic syndrome, cardiovascular disease, chronic kidney disease (CKD), gout, and urolithiasis are associated with hyperuricemia (2). Hyperuricemia is also strongly associated with hypertension, which is the greatest risk factor for stroke and may have an important impact on stroke incidence. In contrast to hyperuricemia, there is no clear definition of hypouricemia; however, a serum uric acid level of 2.0 mg/dL or less is generally used as the reference value (3). Hypouricemia is commonly observed in patients with several neurological diseases, including neurodegenerative diseases. Many epidemiological studies have indicated a link between hypouricemia and an increased risk of developing neurological diseases, suggesting that uric acid may exert neuroprotective effects as an antioxidant (4). Nevertheless, patients with neurological diseases are often undernourished, and hyponutrition may cause hypouricemia. Thus, the causal relationship between uric acid levels and neurological diseases needs to be better understood. When considering brain and neurological diseases, we must consider the conflicting dual nature of uric acid: It has both a vascular risk factor aspect and a neuroprotective aspect. Thus, the effects of uric acid in neurological diseases are likely to vary greatly between stroke, which is a vascular disease, and Parkinson's and Alzheimer's diseases, which are neurodegenerative diseases. Individuals with low uric acid levels are more likely to develop neurodegenerative diseases, whereas hyperuricemia is a risk factor for stroke. Together, these findings indicate that the impact of uric acid is likely to vary depending on the characteristics of the neurological disease (Figure 1). In the present review, we provide evidence for the involvement of uric acid in neurological diseases—including stroke, neuroimmune diseases, and neurodegenerative diseases—and suggest that maintaining appropriate uric acid levels will facilitate future management decisions for neurological diseases.

## 2. Methods

A literature search from August 2022 to November 2022 was performed for this narrative review using PubMed/Medline. English-language articles were identified using the term “uric acid” combined with the terms “neurological diseases,” “neurodegenerative diseases,” “stroke,” “dementia,” “Parkinson's disease,” “amyotrophic lateral sclerosis,” “neuromyelitis optica,” and “multiple sclerosis.”

## 3. Pathogenic role of uric acid in oxidative stress and its neuroprotective effects as an antioxidant

During evolution, mutations in genes involved in ascorbic acid synthesis resulted in their loss of function, and the antioxidant that replaced ascorbic acid was replaced by uric acid because of mutations in uricase. This replacement may be advantageous for survival because ascorbic acid is limited by its propensity for oxidation, producing oxygen radicals and associated mutagens (4). Uric acid and ascorbic acid are considered the most important water-soluble antioxidants, and the plasma uric acid level is approximately six times that of ascorbic acid (5). In addition, Yeum et al. measured antioxidant capacity and found that urate accounted for the majority of the total antioxidant capacity in plasma. This finding suggests that uric acid may be a major endogenous defense against oxidative damage in the body (6). Uric acid can scavenge free radicals under hydrophilic conditions and inhibits lipid peroxidation; however, it loses its antioxidant ability under hydrophobic conditions (7).

## 4. Uric acid regulation in the brain

Xanthine oxidoreductase metabolizes hypoxanthine to xanthine and then to uric acid. Uric acid excretion is performed by several uric acid transporters; approximately two-thirds is excreted by the kidneys, and one-third is excreted by the small intestine. In the brain, xanthine oxidoreductase levels are very low or absent. Thus, very little uric acid is produced in the brain. In addition, uric acid does not penetrate the blood–brain barrier or travel to the brain *via* any uric acid transporters, indicating that uric acid levels in the brain are much lower than those in the blood. Although there is a correlation between the blood and cerebrospinal fluid (CSF) levels, uric acid levels in the CSF in healthy individuals are reportedly only 5–6% of those in the blood. It has therefore been questioned whether uric acid can have any meaningful effect on brain cells (8).

## 5. Uric acid and stroke

According to data from the U.S. National Health and Nutrition Examination Survey (NHANES) obtained in 2015–2018, the prevalence of cardiovascular disease (including coronary artery disease, heart failure, stroke, and hypertension) among adults aged 20 years and older is 49.2% overall (126.9 million in 2018) and increases with age in both men and women (9). Stroke is the second most common cause of death in the world. Ischemic stroke is defined as necrosis of nerve cells because of the sudden cessation of blood in the brain, ultimately leading to brain inflammation (10). Stroke is clinically classified as lacunar stroke, atherosclerotic brain infarction is caused by atherosclerosis, cardiogenic stroke is caused by embolism, and other stroke types such as embolic stroke are caused by an undetermined source. The risk factors common to other cardiovascular diseases, such as hypertension, diabetes, dyslipidemia, hyperuricemia, CKD, and smoking, are involved in the development of stroke. Hyperuricemia may also play a role in the development of cardiovascular disease, stroke, and death; however, insufficient evidence has been provided to date.

### 5.1. Uric acid as a risk factor for stroke

In 2009, Kim et al. reported a systematic review and meta-analysis of the association between hyperuricemia and stroke incidence and death. In this study, hyperuricemia was associated with the risk of both stroke incidence and death, and an analysis adjusting for known risk factors such as age, hypertension, diabetes, and cholesterol also found a similar association (11). In 2014, Li et al. reported a meta-analysis of the association between hyperuricemia and stroke incidence and mortality and similarly found that hyperuricemia was correlated with both stroke incidence and mortality (12). This relationship was more pronounced in women. In 2017, a meta-analysis revealed a significant dose–response relationship between increased serum uric acid levels and stroke risk, with an ~10% increase in stroke risk for every 1 mg/dL increase in serum uric acid levels (13). In addition, the Reasons for Geographic and Racial Differences in Stroke (REGARDS) study in 2020 was a case–cohort study with a large data set. This study also concluded that hyperuricemia may be a risk factor for stroke and noted that hypertension severity may be a confounding factor in the association between serum uric acid levels and stroke (14). Together, these findings suggest that hyperuricemia is strongly associated with hypertension and may have a significant impact on stroke incidence. However, because negative results also exist regarding the relationship between serum uric acid levels and stroke, serum uric acid levels may not be a clear risk factor for stroke (15–17). These inconsistent results may derive from differences in study settings and participants. Table 1 summarized the evidence of investigating the relationship between stroke and serum uric acid levels (11–17, 22, 23).

**Table 1:** Evidence of investigating the relationship between stroke and serum uric acid levels.

**Study**	**Subjects**	**Study design**	**Main results**	**Conclusion**
Verdecchia et al. (22)	1,720 participants with essential hypertension	Cohort study (prospective cohort study)	CVD events (RR 1.73, 95% CI, 1.01–3.00) All-cause mortality (RR 1.63, 95% CI 1.02–2.57)	Elevated uric acid is a risk for CVD and all-cause mortality.
Kim et al. (11)	238,449 participants (16 studies)	Systematic review and meta-analysis	Stroke incidence (6 studies; RR 1.41, 95% CI 1.05–1.76) Mortality (6 studies; RR 1.36, 95% CI 1.03–1.69)	Hyperuricemia may modestly increase the risks of both stroke incidence and mortality.
Li et al. (12)	22,571 stroke patients and 1,042,358 participants (15 prospective studies)	Meta-analysis	Stroke incidence (RR 1.22, 95% CI 1.02–1.46) Mortality (RR 1.33, 95% CI 1.24–1.43)	Hyperuricemia may increase the risks of both stroke incidence and mortality.
Zhang et al. (15)	36,313 participants (20,685 women and 15,628 men)	13 cohort studies	CVD mortality (HR 1.51, 95% CI 1.14–1.99) in women CVD mortality (HR 1.28, 95% CI 1.01–1.63) in men	J- or U-shaped relationship between serum uric acid levels and cardiovascular mortality.
Kamei et al. (23)	155,322 participants (94,746 women and 60,576 men)	Cohort study	Nonfatal stroke incidence (OR 1.24, 95% CI 1.00–1.48) in women Nonfatal stroke incidence (OR 1.26, 95 % CI 1.04–1.54) in men	Serum uric acid is independently associated with the incidence of nonfatal stroke.
Zhong et al. (13)	10,229 stroke patients and 359,243 participant	Meta-analysis	Stroke incidence for an increase in uric acid levels of 1 mg/dl (RR 1.11 95% CI 1.09–1.13) in women	Elevated serum uric acid levels were significantly associated with modestly increased risk of stroke
	12,494 stroke patients and 428,287 participants (11 prospective cohort studies)		Stroke incidence for an increase in uric acid levels of 1 mg/dl (RR 1.10, 95% CI 1.05–1.14) in men	
Chaudhary et al. (14)	951 participants	Case-cohort study	Stroke incidence (HR 1.40, 95%CI 1.10–1.78)	Hyperuricemia may be a risk factor for stroke.
Li et al. (17)	134,20 participants (8,185 women and 5,235 men)	Cohort study (Prospective)	Total stroke (HR 1.45, 95% CI 1.07–1.96) in women Total stroke (HR 1.02, 95% CI 0.74–1.35) in men	Elevated serum uric acid level is an independent predictor of total stroke in women but not in men.
Zhang et al. (16)	9,632 acute ischemic stroke patients (10 studies)	Systematic review and meta-analysis	Vascular events (OR 0.86, 95% CI 0.52–1.41) Mortality (OR 1.08, 95% CI 0.93–1.24)	There was no significant correlation between serum uric acid level and prognosis of ischemic stroke.

RR, relative risk; CI, confidence interval; CVD, cardiovascular disease; OR, odds ratio; HR, hazard ratio.

### 5.2. Stroke and hyperuricemia associated with CKD

In CKD, uric acid excretion decreases, and serum uric acid levels increase as the glomerular filtration rate decreases. Although CKD is considered an independent risk factor for stroke, there are no clear reports on the association between uric acid levels and stroke in CKD patients. However, treatment with allopurinol, which inhibits uric acid synthesis by blocking xanthine oxidase activity, has been reported to reduce the progression of CKD (18). In addition, allopurinol treatment is associated with lower rates of stroke in older patients with hypertension, particularly at higher doses of allopurinol (19). However, because no prospective clinical trials have evaluated whether allopurinol improves stroke, there is not yet sufficient evidence for its use in stroke patients.

CKD may also increase the risk of stroke in patients with non-valvular atrial fibrillation. A meta-analysis by Zeng et al. showed that atrial fibrillation patients with an estimated glomerular filtration rate of less than 60 mL/min had a significantly higher risk of developing thromboembolic events than patients with an estimated glomerular filtration rate of over 60 mL/min (relative risk: 1.62, 95% confidence interval: 1.40 to 1.87, *p* < 0.001). The annual incidence of thromboembolic events increased by 0.41% (95% confidence interval: 0.17% to 0.65%) with a 10 mL/min decrease in renal function (20). Furthermore, Singh et al. demonstrated an association between allopurinol use and the risk of incident atrial fibrillation in the elderly. In a multivariate-adjusted analysis, allopurinol use was associated with the development of atrial fibrillation (hazard ratio: 0.83, 95% confidence interval: 0.74 to 0.93). In addition, prolonged allopurinol use was associated with a lower hazard ratio for atrial fibrillation (21).

### 5.3. Hypouricemia and stroke

Several epidemiologic studies have shown a J-shaped association between serum uric acid and cardiovascular events. In the Progetto Ipertensione Umbria Monitoraggio Ambulatoriale (PIUMA) study of a large cohort of patients with essential hypertension, the relationship between serum uric acid and cardiovascular events and all-cause mortality was J-shaped in both men and women, with a nadir in the second quartile (4.5 to 5.2 mg/dL in men, 3.2 to 3.9 mg/dL in women). These results indicate that cardiovascular events increase with hypouricemia (≤ 4.5 mg/dL in men, ≤ 3.2 mg/dL in women). (22). Moreover, in a longitudinal national cohort study by Kamei et al., serum uric acid levels above 7.1 mg/dL in men and 5.5 mg/dL in women were independently associated with the incidence of non-fatal stroke. Similarly, the risk of stroke was increased in this study, even in the group with lower uric acid levels, with a J-shaped curve (23). In other studies, the relationship between serum uric acid levels and stroke risk differed by sex, with a J-shaped relationship in men but a nearly linear trend in women (13, 15). Thus, although a wellestablished relationship between serum uric acid levels and stroke seems to exist, a causal relationship remains controversial because of a lack of solid evidence (24).

### 5.4. Uric acid localization in vessels

Using carotid endarterectomy specimens, Patetsios et al. found the presence of uric acid, along with cholesterol and xanthine oxidase, in atherosclerotic plaques (25). In addition, Park et al. found that monosodium urate (MSU) was present in the coronary arteries of gout patients. They suggested that uric acid crystals in the intima of blood vessels in gout patients may cause inflammation that leads to plaque rupture, thereby posing a risk of cardiovascular disease (26). This mechanism is thought to be the same mechanism by which gouty arthritis develops. In gouty arthritis, activation of gout salt microcrystals induces multiple inflammatory mediators, including interleukin-1β in the NALP3 inflammasome pathway (27). Similarly, in blood vessels, MSU crystal deposits in coronary arteries can trigger a corresponding inflammatory cascade leading to thrombosis and infarction. Several studies have used dual-energy computed tomography to reveal intravascular MSU deposits. For example, Klauser et al. identified MSU deposits in vessels by polarized light microscopy and dual-energy computed tomography; intravascular MSU deposition was detected frequently in gout patients and was associated with higher coronary calcium scores (28).

### 5.5. Effects of uric acid-lowering drugs in stroke

Hyperuricemia is associated with stroke events, but it is necessary to evaluate whether the drug-induced reduction of uric acid decreases the risk of these events. Larsen et al. conducted a cohort study of patients with serum uric acid levels of 6 mg/dL or higher in Denmark from 1992 to 2010. Among the 65,971 hyperuricemic patients surveyed, 7,127 patients were taking allopurinol. Compared with non-users, allopurinol users had a hazard ratio of 0.89 (95% confidence interval: 0.81 to 0.97) for cardiovascular outcomes and a hazard ratio of 0.68 (95% confidence interval: 0.62 to 0.74) for all-cause mortality. These results indicate that oral allopurinol is associated with a risk reduction of major cardiovascular events, including stroke (29). Moreover, in a study examining the association between allopurinol treatment initiation and stroke from 2006 to 2012, allopurinol treatment reduced the hazard ratio for stroke by 9% (95% confidence interval: 0.83 to 0.99). The beneficial effect of starting allopurinol treatment was seen at 6 months after initiation and was greater with longer use (30). MacIsaac et al. followed cardiovascular outcomes for 10 years in hypertensive patients aged 65 years and older in the United Kingdom. Allopurinol reduced the hazard ratio for stroke by 0.50 (95% confidence interval: 0.32 to 0.80) and for cardiovascular events by 0.61 (95% confidence interval: 0.43 to 0.87); this result was particularly true for high doses (300 mg/day or higher) (19).

A few prospective trials have also been conducted in patients with acute ischemic stroke. In a randomized, double-blind, controlled study, 70 patients (45 women and 25 men) with acute ischemic stroke received allopurinol (200 mg/day) for 3 months and were evaluated for functional outcomes using the modified Rankin scale. After 3 months, the rate of good functional status (modified Rankin scale score = 0–2) was 40.0% in the placebo group and 65.7% in the allopurinol group. A strong association was observed between good functional status and allopurinol administration (odds ratio = 4.646, *p* = 0.014) (31). By contrast, in a large observational study that compared probenecid with allopurinol in patients aged 65 years or older with gout, the gout patients initiating treatment with probenecid had a lower risk of hospitalization for myocardial infarction or stroke than those treated with allopurinol (hazard ratio: 0.80, 95% confidence interval: 0.69 to 0.93). These results thus indicate no beneficial effects of allopurinol (32). Similarly, the Febuxostat Vs. Placebo Randomized Controlled Trial Regarding Reduced Renal Function in Patients With Hyperuricemia Complicated by Chronic Kidney Disease Stage 3 (FEATHER) study—a multicenter, randomized, controlled trial in Japan—reported no difference in all-cause mortality, cardiac disease, or stroke events after 108 weeks of treatment with febuxostat vs. placebo (33). The multicenter, prospective, randomized, open-label Febuxostat for Cerebral and CaRdiorenovascular Events PrEvEntion StuDy (FREED) trial also showed no difference in the primary composite endpoint (death from cerebral, cardiovascular, or renal disease) between the febuxostat and non-febuxostat groups at 36 months of observation (34). Table 2 summarized the evidence for pharmacological intervention with uric acid lowering drugs for stroke (29–34).

**Table 2:** Evidence for pharmacological intervention with uric acid lowering drugs for stroke.

**Study**	**Subjects**	**Study design**	**Intervention**	**Main results**	**Conclusion**
Larsen et al. (29)	65,971 patients with high uric acid levels (≥6 mg/dL)	Cohort study	Allopurinol	Composite outcome (MI, stroke, or CV death) (HR 0.89, 95% CI 0.81–0.97) All-cause mortality (HR 0.68, 95% CI 0.62–0.74)	Allopurinol is associated with lower CV risk among patients with hyperuricemia.
Singh and Yu (30)	28,488 patients of allopurinol use	Cohort study	Allopurinol	Stoke incidence (HR 0.91, 95 % CI 0.83–0.99).	Allopurinol is associated with lower risk of stroke.
Taheraghdam et al. (31)	70 patients (45 females, 25 males) with acute ischemic stroke who had elevated levels of serum uric acid	Randomized, double-blind, controlled study	Allopurinol (200 mg/day) vs. placebo	Modified Rankin scale = 0–2 was 23 (65.7%) in allopurinol and 14 (40.0%) in placebo (OR 4.646, *p* = 0.014)	Allopurinol is improved the 3-month functional status of patients with acute ischemic stroke who had high levels of serum uric acid.
Kim et al. (32)	9,722 patients with gout	Cohort study	Probenecid or allopurinol	Hospitalization for MI or stroke. MI was 2.36 in probenecid and 2.83 in allopurinol (HR 0.80, 95% CI 0.69–0.93)	Probenecid is associated with lower risk of CV events including MI, stroke compared with allopurinol.
Kimura et al. (33)	467 patients with stage 3 CKD and asymptomatic hyperuricemia	Randomized, double-blind, placebo-controlled trial	Febuxostat or placebo	Stroke incidence was 2 patients in placebo and 1 patients in febuxostat	No difference in stroke events with febuxostat vs. placebo.
Kojima et al. (34)	1,070 elderly patients with hyperuricemia (serum uric acid >7.0 to ≤ 9.0 mg/dl)	Randomized, open-label, blinded endpoint study	Febuxostat or non-febuxostat	Cerebrovascular disease was 1.7% in febuxostat and 1.3% in non-febuxostat (HR 1.271, 95% CI 0.479–3.371)	Febuxostat did not reduce the incidence of cerebrovascular disease in patients with hyperuricemia.

CV, cardiovascular; HR, hazard ratio; CI, confidence interval; OR, odds ratio; MI, myocardial infarction: CKD, chronic kidney disease.

## 6. Parkinson's disease

Parkinson's disease is the second most common neurodegenerative disease after Alzheimer's disease and is the most common neurodegenerative disease-causing movement disorder. The incidence of new onset is 5–35 per 1,00,000 persons, and the prevalence is 100–300 per 1,00,000 persons (35). The most common age of Parkinson's disease onset is 50–65 years, but the incidence usually increases with advancing age. Parkinson's disease is generally twice as common in men as in women, although some reports in Japan suggest that it is somewhat more common in women (36). Parkinson's disease is characterized by the loss of neurons in specific areas of the substantia nigra as well as extensive intracellular protein (alpha-synuclein) accumulation (35). Clinical diagnosis is made by identifying characteristic motor signs such as resting tremor and bradykinesia. Additionally, non-motor symptoms such as olfactory impairment, constipation, sleep disturbances, and cognitive impairment commonly occur at all stages of the disease.

### 6.1. Uric acid levels in Parkinson's disease

Elevated blood uric acid levels have been linked to a decreased risk of developing Parkinson's disease. Analyses adjusted for age, smoking, and caffeine use showed that higher uric acid quartiles were associated with lower odds ratios for Parkinson's disease. The odds ratio for the highest quartile compared with the lowest quartile was 0.43 (95% confidence interval: 0.18 to 1.02). A significant dose–response relationship was also observed when uric acid levels were analyzed as a continuous variable. The odds ratio for Parkinson's disease decreased by 0.76 for every 1 mg/dL increase in uric acid concentration (95% confidence interval: 0.61 to 0.95; *p* = 0.017) (37).

The Parkinson Research Examination of CEP-1347 Trial (PRECEPT), including 84 patients with early-stage Parkinson's disease (mean follow-up 21.4 months), was conducted from April 2002 to August 2005 to evaluate the efficacy of neuroprotective agents on the rate of Parkinson's disease progression. The primary outcome was progression to clinical disability requiring dopaminergic therapy. Parkinson's disease progression decreased with increasing serum uric acid levels; this association was stronger in men than in women. This study also evaluated changes in striatal uptake of the presynaptic dopamine transporter marker, iodine I 123-labeled 2-beta-carbomethoxy-3-beta-(4-iodophenyl) tropane. The rate of loss of this uptake also improved with increasing serum uric acid levels (38). Similarly, in the Deprenyl and Tocopherol Antioxidative Therapy of Parkinsonism (DATATOP) study, higher baseline serum and CSF uric acid levels were linked to a slower progression of Parkinson's disease (39).

Moccia et al. showed a relationship between serum uric acid levels and the presence and progression of non-motor symptoms in new-onset Parkinson's disease patients (40). In drug-naïve patients with Parkinson's disease, serum uric acid levels were correlated with the severity of dopaminergic impairment in the caudate, putamen, and striatum on dopamine transporter scans (41). Sleeman et al. assessed movement severity with the Movement Disorder Society Unified Parkinson's Disease Rating Scale Part III and demonstrated that lower serum uric acid levels were associated with worse motor function in patients with Parkinson's disease (42). These results were reviewed, and lower plasma uric acid levels in men were added as a new biomarker in the Movement Disorder Society study criteria for prodromal Parkinson's disease (43), a condition in which patients do not yet exhibit typical motor signs. Together, these findings suggest a strong epidemiological association between Parkinson's disease and uric acid; low serum uric acid is associated with the risk of development, progression, and severity of Parkinson's disease.

Although the exact pathogenesis of Parkinson's disease is still unclear, oxidative stress because of mitochondrial dysfunction, neuroinflammation, and alpha-synuclein misfolding/aggregation and impaired protein clearance triggered by multifactorial factors such as aging, genetic factors, and environmental/lifestyle factors could be involved (44). Uric acid is a potent antioxidant and is considered a potential neuroprotective agent. If healthy individuals have neuroprotection through uric acid, older individuals who develop Parkinson's disease may lack this defense and be more vulnerable to oxidative stress (45); *in vitro* and *in vivo* studies have shown that uric acid protects dopaminergic neurons (4). The prevailing view is that low uric acid levels increase oxidative stress, which causes dopaminergic neuron degeneration and triggers disease onset. Meanwhile, reverse causality suggests that symptoms and conditions associated with Parkinson's disease—such as mitochondrial dysfunction, inherently low purine metabolism, dietary habits, changes in gastrointestinal motility and microbiomes, and reduced physical activity—may lead to low uric acid levels (8). Table 3 summarized the evidence of investigating the relationship between Parkinson's disease and serum uric acid levels (37–42).

**Table 3:** Evidence of investigating the relationship between Parkinson's disease and serum uric acid levels.

**Study**	**Subjects**	**Study design**	**Main results**	**Conclusion**
Weisskopf et al. (37)	18,018 participants	Cohort study	Rate ratio of Parkinson's disease (HR 0.43, 95% CI 0.18–1.02)	High plasma uric acid levels may decrease the risk of Parkinson's disease
Schwarzschild et al. (38)	804 patients with early Parkinson's disease	Cohort study (Prospective)	Progression to clinical disability (HR 0.51, 95% CI, 0.37–0.72)	Serum uric acid linked to the progression of Parkinson's disease.
Ascherio et al. (39)	800 subjects with early Parkinson's disease	Double-blind, randomized trial	Progression to clinical disability (HR 0.64, 95% CI 0.44–0.94)	Higher serum uric acid levels were associated with slower rates of clinical decline.
Moccia et al. (40)	69 newly diagnosed Parkinson's disease patients	Cohort study (Prospective)	Overall non-motor symptoms progression (OR 0.488, *p* = 0.023)	Serum uric acid is associated with presence and progression of multiple non-motor symptoms in newly diagnosed Parkinson's disease.
Moccia et al. (41)	52 newly diagnosed Parkinson's disease patients	Cohort study	Uric acid levels were significantly higher in patients with higher averaged, dopamine transporter binding.	Uric acid levels correlates with the severity of dopaminergic impairment.
Sleeman et al. (42)	154 newly diagnosed Parkinson's disease patients and 99 age-matched controls	Cohort study (Prospective)	The interaction of uric acid with time was a predictor of change in Movement Disorders Society Unified Parkinson's Disease Scale Part III score.	Lower serum uric acid level is associated with worsening motor function.

HR, hazard ratio; CI, confidence interval; OR, odds ratio.

### 6.2. Inosine treatment in Parkinson's disease

Oral administration of uric acid cannot increase serum uric acid levels because uric acid is rapidly degraded by the bacterial flora in the intestinal tract. However, oral ingestion of its precursor, inosine, quickly increases serum urate levels (46). Therefore, a randomized, double-blind, placebo-controlled, dose-ranging study of inosine (Safety of Urate Elevation in Parkinson's Disease [SURE-PD]) was conducted. Seventy-five outpatients attending an American Parkinson Study Group-accredited clinical trial facility between 2009 and 2011 were enrolled and received a placebo or inosine (up to two 500 mg capsules orally, up to three times a day for up to 24 months, median 18 months) to produce mildly (6.1–7.0 mg/dL) or moderately (7.1–8.0 mg/dL) high serum uric acid levels. The primary endpoints were the absence of unacceptable serious adverse events (“safety”), continued treatment without adverse events requiring dose reduction (“tolerability”), and elevated serum and CSF uric acid levels. Inosine was shown to be safe, well tolerated, and effective in increasing serum and CSF uric acid levels (47). In addition, Bhattacharyya et al. found a dose-dependent increase in serum uric acid levels with an increase in antioxidant capacity as assessed using urine 8-hydroxydeoxyguanosine (48). These results prompted a randomized, double-blind, placebo-controlled, phase III study of oral inosine in early Parkinson's disease. The primary endpoint was the rate of change in the Movement Disorders Society Unified Parkinson's Disease Rating Scale prior to the initiation of dopaminergic therapy; however, the rate of parkinsonism progression did not differ between the placebo and inosine groups (49). Although this study included Parkinson's disease patients who would be expected to benefit more from elevated uric acid treatment, the increase in uric acid levels with inosine did not seem to support a protective effect of uric acid. This may be because the urate precursor inosine, rather than uric acid, was received; this may have had detrimental effects that offset the benefits of uric acid elevation, such as mediating the production of xanthine oxidase. However, it may also be that uric acid is more useful as a biomarker for the severity and progression of Parkinson's disease than as a neuroprotective agent. Table 4 summarized the evidence of investigating the relationship between receiving inosine and neurodegenerative diseases (38, 47, 48, 59–62, 75).

**Table 4:** Evidence of investigating the relationship between receiving inosine and neurodegenerative diseases.

**Study**	**Subject**	**Study design**	**Main results**
Bhattacharyya et al. (48)	Early Parkinson's Disease	Randomized, placebo-controlled trial	Inosine has a increase in plasma antioxidant capacity.
Schwarzschild et al. (47)	Early Parkinson's Disease	Randomized, double-blind, placebo-controlled trial	Inosine may slow clinical disease progression in women.
Schwarzschild et al. (38)	Early Parkinson's Disease	Randomized, double-blind, placebo-controlled trial	Inosine did not slow clinical disease progression.
Toncev et al. (59)	Multiple sclerosis	Clinical trial	
Markowitz et al. (60)	Relapsing-remitting multiple sclerosis	Randomized, double-blind trial	Inosine may have benefits for at least some multiple sclerosis patients.
Gonsette et al. (61)	Relapsing-remitting multiple sclerosis	Randomized, double-blind, placebo-controlled trial	Inosine was not additional advantage.
García et al. (62)	Relapsing-remitting multiple sclerosis	Randomized, double-blind, placebo-controlled trial	Inosine was not effective in patients with Relapsing-remitting multiple sclerosis.
Nicholson et al. (75)	Amyotrophic lateral sclerosis	Open-label trial	Inosine improved biomarkers of oxidative stress and its damage.

## 7. Multiple sclerosis

Multiple sclerosis is a chronic inflammatory demyelinating disease that affects the central nervous system and is most frequently associated with disability in young adults. Multiple sclerosis affects an estimated 2.8 million people worldwide but has a heterogeneous prevalence. The majority of multiple sclerosis patients are diagnosed between the ages of 20 and 50 years, but the disease can develop at any age. The incidence of disease is higher in women than in men. The cause of multiple sclerosis is multifactorial, with both genetic and environmental risk factors contributing to disease risk. In terms of genetic risk, the association between HLA-DRB1^*^15:01 and a high risk of multiple sclerosis has been known for decades. The most commonly considered environmental risk factor is infection with Epstein–Barr virus; other factors that may be involved include smoking, vitamin D levels, ultraviolet light, vaccines, and obesity (50). However, the specific causes of multiple sclerosis remain largely unknown, and there are currently no wellestablished factors that can help to prevent the disease.

### 7.1. Uric acid levels in multiple sclerosis

A meta-analysis by Wang et al. showed that serum uric acid levels in patients with multiple sclerosis were significantly lower than in healthy individuals, which supports an association between multiple sclerosis and uric acid (51). Moreover, Drulović et al. reported that serum uric acid levels tended to be lower in multiple sclerosis patients than in patients with other neurological diseases although this difference was not significant (*P* = 0.068). However, the mean serum uric acid level in active multiple sclerosis patients was significantly lower than that in inactive multiple sclerosis patients (*P* = 0.046) and patients with other neurological diseases (*P* = 0.007). These results indicate that serum uric acid might act as a biomarker of disease activity in multiple sclerosis (52). Supporting this idea, Simental-Mendía et al. suggested that elevated uric acid levels are likely linked to the presence of benign multiple sclerosis (53). Furthermore, in patients with relapsing-remitting multiple sclerosis, Moccia et al. reported a link between the progressive reduction of uric acid levels and relapse risk, disability progression, and cognitive function (54). They also examined serum uric acid levels in patients with relapsing-remitting and secondary-progressive forms of multiple sclerosis and healthy individuals; their findings suggested the importance of serum uric acid as a biomarker of multiple sclerosis disability and progression (55). Niu et al. performed a meta-analysis of 110,347 patients; their estimate based on the single-nucleotide polymorphism rs12498742, which explained the largest proportion of variance, revealed that the odds ratio of uric acid for multiple sclerosis was 1.00 (95% confidence interval: 0.90 to 1.11; *p* = 0.96). Mendelian randomization analysis showed that the pooled odds ratio was 1.05 (95% confidence interval: 0.92 to 1.19; *p* = 0.50). The findings of this study, therefore, did not indicate that serum uric acid levels are causally associated with the risk of multiple sclerosis (56).

*In vivo* studies of multiple sclerosis have demonstrated that inosine or inosinic acid can increase serum uric acid levels and has good efficacy (57). Furthermore, oral administration of uric acid did not increase serum uric acid levels, but oral administration of its precursor, inosine, increased serum uric acid levels and was generally well tolerated (58). Toncev conducted a clinical study to evaluate the safety and efficacy of oral inosine as a monotherapy in patients with multiple sclerosis. A total of 32 patients with multiple sclerosis were administered 1–2 g of inosine daily; inosine administration was associated with a significantly lower recurrence rate (*P* = 0.001) and a greater increase in mean Expanded Disability Status Scale (EDSS) scores (*P* = 0.025) (59). Markowitz et al. conducted a randomized, double-blind trial evaluating relapse rate, disability assessed by EDSS, and magnetic resonance imaging features in 16 patients with relapsing-remitting multiple sclerosis who received oral inosine for 1 year. Elevated serum uric acid levels were correlated with fewer gadolinium-enhanced lesions on magnetic resonance imaging as well as improved EDSS. These findings suggest that the use of inosine to increase serum uric acid levels may be beneficial for at least some multiple sclerosis patients (60). Similarly, the ASsociation of Inosine and IFNβ in RRMS (ASIIMS) trial was a multicenter, double-blind, placebo-controlled trial designed to investigate whether inosine supplementation to standard therapy was effective in patients with relapsing-remitting multiple sclerosis. This study did not find any benefits associated with the addition of inosine (61). Muñoz García et al. conducted a trial in which 36 patients with relapsing-remitting multiple sclerosis who started treatment were randomly assigned either inosine (3 g/day) or placebo, with a follow-up period of 12 months. The study concluded that inosine had no neuroprotective role because it had no effect on patients with relapsing-remitting multiple sclerosis. However, they also suggested that phase III trials with a longer follow-up period, of more than 1 year, may be needed to evaluate the efficacy of inosine in preventing disease recurrence and progression (62). Given the results of preclinical and clinical studies of inosine use, the most important properties of inosine seem to be its antioxidant actions and its potential to increase energy resources by improving adenosine triphosphate availability (63). Table 5 summarized the evidence of investigating the relationship between multiple sclerosis and serum uric acid levels (40–42, 51, 52, 56).

**Table 5:** Evidence of investigating the relationship between multiple sclerosis and serum uric acid levels.

**Study**	**Subjects**	**Study design**	**Main results**	**Conclusion**
Drulović et al. (52)	240 multiple sclerosis patients and 104 control patients with other neurological diseases	Cross-sectional study	The mean serum uric acid level was lower in multiple sclerosis, but the difference was not at the level of significance.	Serum uric acid might serve as a marker of disease activity.
Wang et al. (51)	1,537 patients (1,308 multiple sclerosis patients and 229 neuromyelitis optica patients) and 908 healthy participants (10 case-control studies)	Meta-analysis	Serum uric acid levels of patients with multiple sclerosis and neuromyelitis optica were lower (standardized mean difference = −0.52, 95% CI-0.81 to −0.24)	Patients with multiple sclerosis and neuromyelitis optica showed lower serum uric acid levels.
Moccia et al. (40)	362 multiple sclerosis patients and 181 control participants	Cross-sectional study	Multiple sclerosis patients was lower serum uric acid levels (adjusted r^2^ = 0.3036)	Serum uric acid as a biomarker of multiple sclerosis disability and progression
Moccia et al. (41)	141 patients with relapsing-remitting multiple sclerosis	Retrospective longitudinal study	Expanded disability status scale sustained progression (OR 0.099)	Uric acid levels decline progressively in patients with relapsing-remitting multiple sclerosis in relation to relapse risk, disability progression, and cognitive function.
Simental-Mendía et al. (42)	106 multiple sclerosis patients (39 with benign multiple sclerosis patients and 67 with other varieties of multiple sclerosis patients)	Cross-sectional study	Association with the presence of benign multiple sclerosis (OR 2.60; 95% CI 1.55–4.38)	Elevated uric acid levels are linked to the presence of benign multiple sclerosis.
Niu et al. (56)	110,347 multiple sclerosis patients	Meta-analysis	Pooled OR of uric acid for multiple sclerosis was 1.05 (95% CI 0.92–1.19)	Serum uric acid level is causally associated with the risk of multiple sclerosis.

OR, odds ratio; CI, confidence interval.

## 8. Neuromyelitis optica spectrum disorder

Neuromyelitis optica spectrum disorder (NMOSD) is an inflammatory demyelinating disease of the central nervous system that primarily involves the optic nerve and spinal cord (64). After the identification of the aquaporin-4 immunoglobulin G antibody, NMOSD was differentiated from multiple sclerosis. NMOSD-related brain lesions, such as those in the thalamus, periependymal surfaces of the third ventricle, hypothalamus, dorsal medulla, and area postrema, have also been described. In the absence of spinal cord involvement or optic neuropathy, the diagnosis of NMOSD can be established by the presence of aquaporin-4 antibody positivity and NMOSD-associated brain lesions.

### 8.1. Uric acid levels in NMOSD

Several studies have suggested that uric acid and NMOSD are associated. Shu et al. reported that CSF uric acid levels in NMOSD were significantly higher than in control individuals (with non-inflammatory and non-neurodegenerative diseases), but that serum uric acid levels were not significantly different between the two groups. Additionally, they showed that CSF uric acid levels were higher in patients with relapsing NMOSD than in those with NMOSD in the inactive phase. Furthermore, CSF uric acid levels were significantly higher in patients with an impaired blood–brain barrier than in those with an intact blood–brain barrier, suggesting that CSF uric acid levels are affected by blood–brain barrier integrity and may be used as a marker of disease activity (65). In contrast, another study found that serum uric acid levels in NMOSD patients during relapses were significantly lower than those in healthy individuals and that these reduced uric acid levels during NMOSD relapse were normalized during remission (66). In addition, in the differentiation of myelin oligodendrocyte glycoprotein antibody disease and NMOSD, serum uric acid is higher in myelin oligodendrocyte glycoprotein antibody disease, suggesting that serum uric acid may be a potential marker to differentiate these two diseases (67). Liu et al. reported that serum uric acid levels were correlated with NMOSD disease duration and inversely correlated with disability scores, suggesting that serum uric acid may be a useful biomarker for monitoring disease activity (68). Furthermore, a meta-analysis revealed that, similar to in multiple sclerosis, serum uric acid levels are lower in NMOSD patients than in healthy individuals; serum uric acid may thus be a potential diagnostic biomarker for both multiple sclerosis and NMOSD (51). Table 6 summarized the evidence of investigating the relationship between NMOSD and uric acid levels (51, 65–68).

**Table 6:** Evidence of investigating the relationship between neuromyelitis optica spectrum disorders (NMOSD) and uric acid levels.

**Study**	**Subjects**	**Study design**	**Main results**	**Conclusion**
Liu et al. (68)	45 patients (12 in men, 33 in women) who were hospitalized for NMOSD	Cross-sectional study	Mean serum uric acid levels were significantly lower in patients with NMOSD compared to those with cerebral infarction.	Serum uric acid may be a useful surrogate marker for monitoring NMOSD activity.
Min et al. (66)	20 patients with NMOSD and 90 control participants	Cohort study (Retrospective)	Serum uric acid levels during relapses in NMOSD were significantly lower compared to healthy individuals.	Serum uric acid levels are associated with clinical disease status in patients with NMOSD.
Wang et al. (51)	1,537 patients (1,308 multiple sclerosis patients and 229 neuromyelitis optica patients) and 908 healthy participants (10 case-control studies)	Meta-analysis	Serum uric acid levels of patients with multiple sclerosis and neuromyelitis optica were lower (standardized mean difference = −0.52, 95% CI-0.81 to −0.24)	Patients with multiple sclerosis and neuromyelitis optica showed lower serum uric acid levels.
Shu et al. (65)	38 NMOSD patients and 30 control participants	Cross-sectional study	Cerebrospinal fluid uric acid levels in NMOSD were significantly higher, but serum uric acid levels were not statistically significant.	Cerebrospinal fluid uric acid levels significantly increased in NMOSDs patients, but not serum uric acid levels.
Xie et al. (67)	118 aquaporin 4 antibody-positive patients with first-episode NMOSD and 25 patients with first-episode myelin oligodendrocyte glycoprotein antibody disease	Cohort study (Retrospective)	Patients with myelin oligodendrocyte glycoprotein antibody disease had higher uric acid levels than those with AQP4-positive NMOSD.	Serum uric acid level can help distinguish patients with AQP4-positive NMOSD from those with myelin oligodendrocyte glycoprotein antibody disease.

## 9. Amyotrophic lateral sclerosis

Amyotrophic lateral sclerosis (ALS) is a motor neuron disease that involves both upper and lower motor neurons. It is characterized by progressive degeneration of motor neurons, resulting in dysarthria, dysphagia, tetraplegia, and respiratory impairment. Approximately 90–95% of ALS cases are sporadic. The etiology of ALS is unknown, but oxidative stress and glutamate-mediated toxicity are suggested to be involved. The average life expectancy of patients with ALS is 11.3 years with a ventilator and 4.6 years without a ventilator (69). Therefore, disease-modifying therapies that improve patient prognosis are desperately needed.

### 9.1. Uric acid levels and inosine treatment in ALS

It has been suggested that increased uric acid levels may have a beneficial effect against ALS. Additionally, preclinical studies have reported the neuroprotective function of inosine in this disease. Inosine supplementation in fibroblasts from patients with ALS is bioenergetically favorable and may protect against age-related metabolic dysfunction in ALS (70). A descriptive study comprising patients diagnosed with gout from 2011 to 2018 revealed that ALS prevalence was lower in patients with gout than in those without gout (71). In addition, low uric acid levels are reportedly associated with cognitive decline and mortality in ALS (72, 73). The results of a clinical study including 40 patients who received edaravone for ALS suggested that ALS progression was slower in patients with high baseline uric acid levels and in those whose uric acid levels did not decrease (74). Based on these results, an open-label pilot study was conducted that included 25 patients with ALS. Inosine was titrated to increase serum uric acid levels to 7–8 mg/dL and was administrated for 12 weeks. The primary endpoints were safety and tolerability, while the secondary endpoints were biomarkers of oxidative stress and its damage. The results of this clinical trial showed no serious adverse events and no incidence of urinary tract stones or gout, and the administration of inosine resulted in a significant improvement in biomarkers. Inosine thus appears to be safe, well tolerated, and effective in increasing serum uric acid levels in ALS patients. This observation indicates the potential for a large clinical trial to investigate inosine as a therapy for ALS (75). Table 7 summarized the evidence of investigating the relationship between Amyotrophic lateral sclerosis and serum uric acid levels (71–74).

**Table 7:** Evidence of investigating the relationship between Amyotrophic lateral sclerosis and serum uric acid levels.

**Study**	**Subjects**	**Study design**	**Main results**	**Conclusion**
Kwon et al. (71)	Patients diagnosed with gout	Descriptive study	Amyotrophic lateral sclerosis in gout patients was significantly lower than in the general population.	Increased uric acid may have a protective effect in motor neuron diseases.
Tang et al. (72)	124 amyotrophic lateral sclerosis patients	Cross-sectional study	Patients with amyotrophic lateral sclerosis with cognitive impairment had lower plasma uric acid.	Plasma uric acid might help evaluate the risk of cognitive impairment in amyotrophic lateral sclerosis patients.
Xu et al. (73)	313 sporadic and 16 familial amyotrophic lateral sclerosis patients	A longitudinal cohort study	Serum uric acid levels were inversely associated with the decline rate of the Amyotrophic Lateral Sclerosis Functional Rating Scale per month.	Serum uric acid levels are inversely associated with risk of death (especially in men).
Han et al. (74)	40 amyotrophic lateral sclerosis patients with edaravone	Cohort study (Retrospective)	High serum uric acid level at baseline and low rate of decline in uric acid was associated with slower disease progression.	High uric acid level at baseline and low rate of decline in uric acid may predict slow disease progression in amyotrophic lateral sclerosis patients treated with edaravone.

## 10. Relationship between uric acid levels and risk of dementia (Alzheimer's disease and vascular dementia)

In a prospective study of 4,618 participants aged 55 years or older, Euser et al. found a decreased risk of dementia in patients with high serum uric acid levels after correcting for several cardiovascular risk factors (hazard ratio: 0.89, 95% confidence interval: 0.80 to 0.99) (76). Lu et al. used data from the Health Improvement Network in the United Kingdom to evaluate the impact of gout on the risk of developing Alzheimer's disease in a cohort matched for age, sex, time of entry, and body mass index. In this study, 59,224 patients with gout and 238,805 controls were followed for 5 years; 309 new patients with Alzheimer's disease were found among patients with gout (1.0 per 1,000 person-years), and 1,942 new patients were found among controls (1.5 per 1,000 person-years). These results indicate that gout is inversely associated with the risk of developing Alzheimer's disease and support a possible neuroprotective role for uric acid (77). Du et al. analyzed 24 studies, including 21 case–control studies and 3 cohort studies, and found that patients with Alzheimer's disease had lower uric acid levels than healthy individuals (weighted mean difference = −0.77 mg/dL, 95% confidence interval: −2.28 to −0.36, *p* = 0.0002), and that higher serum uric acid levels were correlated with a lower risk of developing Alzheimer's disease (risk ratio: 0.66, 95% confidence interval: 0.52 to 0.85, *p* = 0.001) (78). Furthermore, Cascalheira et al. found that uric acid was an independent predictor of Alzheimer's disease in a case–control study in Portugal of 19 patients diagnosed with Alzheimer's disease and 36 older adult volunteers without symptoms of memory impairment or neurological diseases (odds ratio: 2.42, *p* < 0.02). They also reported a positive correlation between uric acid and Alzheimer's disease; however, the authors stated that this result was not caused by the antioxidant effect of uric acid but rather because reduced renal function is associated with Alzheimer's disease (79).

Khan et al. conducted a systematic review and meta-analysis examining serum uric acid levels in relation to dementia subtypes such as Alzheimer's disease, vascular dementia, and Parkinson's disease-related dementia. Overall, patients diagnosed with dementia had lower serum uric acid levels than controls; however, differences were observed by dementia type. Alzheimer's disease and Parkinson's disease-related dementia showed a strong association with serum uric acid levels, but no correlation was found in patients with vascular or mixed dementia, suggesting a different relationship with serum uric acid levels, especially in patients with vascular dementia compared with other dementia subtypes (80). Moreover, in a cohort of healthy older adults (Three-City Dijon cohort) with a mean age of 72 years, 1,598 patients were followed for 10 years, and 110 developed dementia (8.2/1,000 person-years). The multivariate hazard ratio of the highest to lowest serum uric acid level was 1.79 (95% confidence interval: 1.17 to 2.73, *p* = 0.007), and this association was stronger for vascular or mixed dementia (hazard ratio: 3.66, 95% confidence interval: 1.29 to 10.41, *p* = 0.015) than for Alzheimer's disease (hazard ratio: 1.55, 95% confidence interval: 0.92 to 2.61, *p* = 0.10) (81). These results indicate that high serum uric acid levels are protective against the development of dementia because of the antioxidant effects of uric acid. However, hyperuricemia promotes arteriosclerosis and adversely affects cognitive function, suggesting that both effects should be considered.

Interestingly, the relationship between serum uric acid levels and cognitive function may be stronger in the early stages of cognitive dysfunction. In a study comparing 58 patients with mild cognitive impairment (MCI) with 57 healthy older adults, serum uric acid levels were significantly lower in MCI patients (292.3 μmol/L) than in healthy older adults (322.5 μmol/L; *p* < 0.05) (82). Furthermore, although the sample size was small, there were significant positive correlations between Mini Mental State Examination scores and serum uric acid levels, including orientation, memory, computation, language, and five visual spatial dimensions (*P* < 0.05). In addition, a cross-sectional survey of 2,102 patients was conducted in Beijing, China. The prevalence rates of hyperuricemia and MCI in this community population were 16.7% and 15.9%, respectively, and prevalence rates increased linearly with age (*P* < 0.001) (83).

## 11. Discussion

The relationship between serum uric acid levels and neurological diseases remains controversial. Regarding the reasons, the hypothesis of a toxic effect in atherosclerotic diseases and a protective effect in neurodegenerative diseases and dementia is currently supported. However, the biological mechanisms underlying these relationships have not yet been firmly established. Low uric acid may be a biomarker of neurological diseases, and the possibility that uric acid alterations are a consequence rather than a cause of neurological disease cannot be ruled out. This possibility is supported by the relatively poor results of treatment with inosine, which elevates uric acid. However, it should also be noted that uric acid is not a biologically inert substance but has a wide range of actions including antioxidant, neurostimulatory, inflammation-inducing, and innate immune response-activating activities. In particular, uric acid has two opposing actions—namely antioxidant and oxidative effects—and it has been reported that both hypouricemia and hyperuricemia can lead to the development of cardiovascular diseases. When assessing uric acid levels in the future, it is, therefore, necessary to consider individuals with high and low uric acid separately, limit target diseases, and promote further research.

## Author contributions

NO, EH, and MO: writing—original draft preparation. HT and KS: writing—reviewing and editing. All authors have read and agreed to the published version of the manuscript.
